# Free-breathing with motion-correction and video projection during cardiac MRI : a paediatric design !

**DOI:** 10.1186/1532-429X-16-S1-P319

**Published:** 2014-01-16

**Authors:** Laurent Bonnemains, Freddy Odille, Aboubaker Cherifi, Pierre-Yves Marie, Cedric Pasquier, Jacques Felblinger

**Affiliations:** 1 Cardiology, CHU Nancy, Nancy, France; 2 INSERM,U947, Nancy, France; 3 Université de Lorraine, Nancy, France; 4 Radiology, CHU Nancy, Nancy, France; 5 CICIT 801, CHU Nancy, Nancy, France

## Background

Cardiac magnetic resonance imaging with children is a complex exam because it requires cooperation of the children to stay motionless and perform multiple breath-holding. This issue is particularly accute for fibrosis detection with late gadolinium enhancement which requires strict immobility during acquisition. In many situations, children must undergo general anaesthesia because such cooperation is too difficult to obtain. However,1/cooperation and stillness is easily obtained when children are proposed funny derivative occupation (such as movies to watch) during the exam and 2/the blurring effect induced by free-breathing can be corrected by a retrospective non-rigid adaptative motion correction algorithm such as GRICS (Odille, MRM 2008).

## Methods

**Figure 1:**
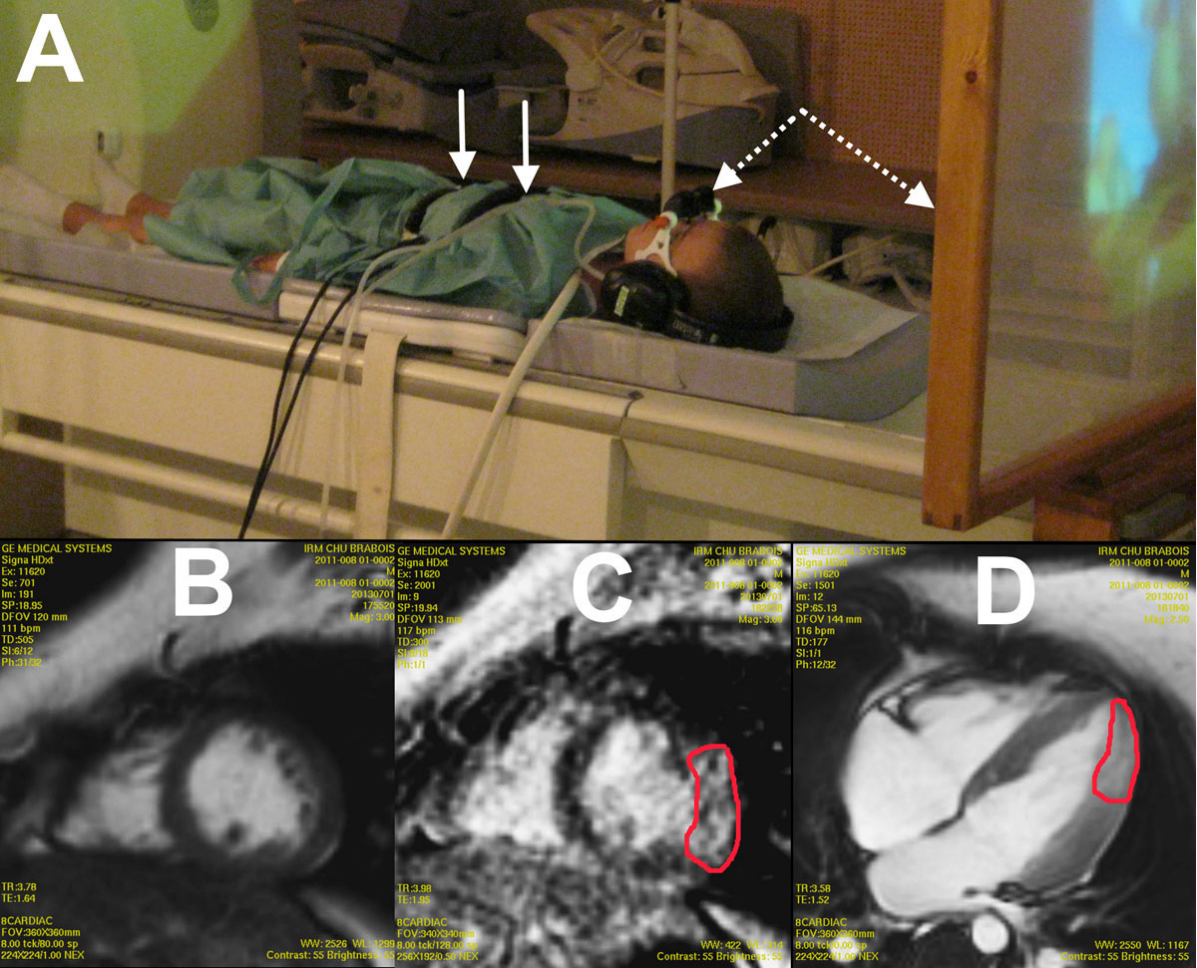
**(A) Example of installation of a child for cardiac MRI examination**. The white arrows indicate the two respiratory belts required by GRICS, whereas the dashed arrows point on the oblique glasses and the wood screen used to project cartoons. (BCD) Examples of images obtained in full free-breathing and reconstructed with GRICS: Two mid-ventricular short-axis views (B: before injection and C:after injection ) and one horizontal long axis view after injection. Diffuse fibrosis can be seen in the laterals segments (inside the contoured zones).

We present an alternative solution based on GRICS (non-rigid adaptative ungated motion correction method) and on simple basic equipment (special glasses, screen, and video projector) as illustrated in Figure 1A. GRICS requires the use of two respiratory belts to record the thorax motions during the examination, in order to correct it during the image reconstruction process. We were able to organize full cardiac MRI examinations (Cine-MRI before and after gadolinium, Phase contrast for aortic flow and myocardial delayed enhancement) with GRICS for four Duchene children (mean = 9 years old) in complete free breathing and with almost no instruction given to the children from the MR operator during the exams.

## Results

Exams were easily performed. Every children found the experience enjoyable. For three of them, the exam revealed diffuse fibrosis in the lateral or infero-lateral segments, as illustrated in Figure 1C and 1D, whereas the images before injection were normal and no alteration of ejection fraction was noted.

## Conclusions

Paediatric complete free-breathing cardiac MRI is feasible with GRICS and simple video equipment. GRICS greatly simplifies the organisation of cardiomyopathy MRI examination with young children. This is the first report of the use of GRICS with patients.

## Funding

Joint funding from INSERM,France and AFM,France (French Association against Myopathy) under the ID C13-04.

